# Cellular Senescence in Sarcopenia: Possible Mechanisms and Therapeutic Potential

**DOI:** 10.3389/fcell.2021.793088

**Published:** 2022-01-10

**Authors:** Yongyu He, Wenqing Xie, Hengzhen Li, Hongfu Jin, Yi Zhang, Yusheng Li

**Affiliations:** ^1^ Department of Orthopedics, Xiangya Hospital, Central South University, Changsha, China; ^2^ Xiangya School of Medicine, Central South University, Changsha, China; ^3^ National Clinical Research Center for Geriatric Disorders, Xiangya Hospital, Central South University, Changsha, China

**Keywords:** aging, sarcopenia, cellular senescence, muscle stem cells (MuSCs) dysfunction, senescence-associated secretory phenotype (SASP)

## Abstract

Aging promotes most degenerative pathologies in mammals, which are characterized by progressive decline of function at molecular, cellular, tissue, and organismal levels and account for a host of health care expenditures in both developing and developed nations. Sarcopenia is a prominent age-related disorder in musculoskeletal system. Defined as gradual and generalized chronic skeletal muscle disorder, sarcopenia involves accelerated loss of muscle mass, strength and function, which is associated with increased adverse functional outcomes and evolutionally refers to muscle wasting accompanied by other geriatric syndromes. More efforts have been made to clarify mechanisms underlying sarcopenia and new findings suggest that it may be feasible to delay age-related sarcopenia by modulating fundamental mechanisms such as cellular senescence. Cellular senescence refers to the essentially irreversible growth arrest mainly regulated by p53/p21^CIP1^ and p16^INK4a^/pRB pathways as organism ages, possibly detrimentally contributing to sarcopenia via muscle stem cells (MuSCs) dysfunction and the senescence-associated secretory phenotype (SASP) while cellular senescence may have beneficial functions in counteracting cancer progression, tissue regeneration and wound healing. By now diverse studies in mice and humans have established that targeting cellular senescence is a powerful strategy to alleviating sarcopenia. However, the mechanisms through which senescent cells contribute to sarcopenia progression need to be further researched. We review the possible mechanisms involved in muscle stem cells (MuSCs) dysfunction and the SASP resulting from cellular senescence, their associations with sarcopenia, current emerging therapeutic opportunities based on targeting cellular senescence relevant to sarcopenia, and potential paths to developing clinical interventions genetically or pharmacologically.

## 1 Introduction

Population statistics according to the World Health Organization (WHO) revealed that population ageing has occurred at an unprecedented speed globally (Rong et al., 2020). Indeed, advanced chronological age is the very notable risk factor for most of the world’s chronic diseases, which are characterized by progressive molecular, cellular, tissue and organic functional decline over time (Boengler et al., 2017). For example, age-associated diseases are well-characterized by the presence of several markers appearing with advanced ageing that are varying degrees shown in all organs. Age-related disorders are responsible for considerable healthcare expenditures in both developing and developed nations (Dennison et al., 2017). Among the elderly, sarcopenia is deemed to be a remarkable hallmark that accrues across a lifetime (Brook et al., 2016), whilst it represents a muscle disease (muscle failure) rooted in adverse muscle changes possibly occurring in progeria. Sarcopenia is closely related to negative outcomes in the elderly, including impaired physical performance: Declined muscle strength resulting in an increased risk of falls and fracture (Yeung et al., 2019). Sarcopenia patients are suffering from progressive decline of muscle function that eventually increases mobility disorders contributing to negative physical outcomes and decreasing their quality of life according to clinical researches (Wiedmer et al., 2021). Recent mounting researches, however, have made remarkable strides in understanding fundamental mechanisms contributing to sarcopenia, and one such modifiable basic aging mechanism that has gained considerable attention in skeletal muscle is cellular senescence (Muñoz-Espín and Serrano, 2014). Specifically, more efforts have focused on the identification of effectors contributing to sarcopenia including impaired mitochondrial function, reducing number of motor units, declined number and regenerative capacity of MuSCs, which cellular senescence in skeletal muscle correlates with (Wiedmer et al., 2021). However, further studies should be conducted to fully understand the mechanisms by which senescent cells contribute to disease progression. Excessive evidence suggests that interventions targeting sarcopenia in association with age-induced cellular senescence are extremely potential, which involve strategies regulating some molecules on the pathway of cellular senescence in sarcopenia, individual nutrition levels such as caloric restriction (Xie et al., 2020b), and individual physical levels including health education and exercise interventions (Xie et al., 2020a). This review aims to delineate the biological roles of cellular senescence in sarcopenia and their causal roles in mediating or delaying sarcopenia, which may activate therapeutic strategies for sarcopenia clinically, eventually extending health span in the elderly population.

## 2 Sarcopenia

Sarcopenia was first described by Rosenberg et al. in 1989 as a predominantly geriatric condition, with a progressive loss of skeletal muscle mass, muscle strength and muscle function with advancing age (Papadopoulou, 2020). Since then it has increasingly become an awareness that an age-related decline in muscle mass and lean body mass contributes to loss of muscle function, associated with increasing possibility in weakness, falls, frailty and fractures (Rosenberg, 1997).

The term sarcopenia was first used to describe the accelerated loss of muscle mass, strength and function. However, a universally accepted definition of sarcopenia has yet been reached for decades. Nevertheless, with sarcopenia gradually being used in other relevant systems apart from skeletal muscle system such as cardiovascular disease (Izumida and Imamura, 2020), cancer (gastric cancer (Sierzega and Chrzan, 2019)), endocrine system (diabetes (Fukuoka et al., 2019), the European Working Group Sarcopenia (EWGSOP)) published progress and updates of the definition of sarcopenia in the Age and Aging, aiming to achieve a consensus definition of it. Cruz-Jentoft et al. recognized sarcopenia as a new geriatric syndrome (Cruz-Jentoft et al., 2010b), though such definition has recently been progressed and updated by the EWGSOP in 2010 (Cruz-Jentoft et al., 2010a), revised by the EWGSOP2 in 2018 (Cruz-Jentoft et al., 2019), and supported by the Asian Working Group on Sarcopenia (AWGS) in 2014 (Chen et al., 2014). A practical clinical definition and consensus diagnostic criteria for age-related sarcopenia was initially developed by the EWGSOP, also endorsed by four participant organizations (the European Geriatric Medicine Society, the European Society for Clinical Nutrition and Metabolism, the International Association of Gerontology and Geriatrics-European Region and the International Association of Nutrition and Aging). The EWGSOP cited a working definition of sarcopenia, the presence of primary muscle mass plus low muscle strength or low physical performance (Cruz-Jentoft et al., 2010a). In order to foster advances in reflecting scientific and clinical evidence of sarcopenia, the EWGSOP2 updated the recommendations of sarcopenia that loss of muscle strength acts as the forefront parameter of sarcopenia, followed by low muscle quality/quantity and low physical performance (Cruz-Jentoft et al., 2019). In operational definition of sarcopenia, considering the appropriate diagnostic values of sarcopenia, the AWGS applied screening tests in measuring both muscle strength (handgrip strength) and physical performance (Chen et al., 2014). Evolutionally, Bauer et al. (2019) categorized sarcopenia into primary sarcopenia, an age-related one, and secondary sarcopenia, a disease-related one, which is accompanied with diabetes mellitus, cancer, chronic obstructive pulmonary disease, or heart failure. Further research is needed to figure out the mechanisms underlying the pathological process of sarcopenia, which may act as the fundament of the subsequent interventions and even clinical trials for sarcopenia.

## 3 Cellular Senescence

Cellular senescence was first described as a phenomenon that human diploid fibro-blasts derived from fetuses proliferated their life span with a limited capacity when cultured by screening the maintenance of the diploid karyotype, retention of sex chromatin, histological differentiation and so on by Leonard Hayflick and Paul Moorhead in 1960s (Hayflick and Moorhead, 1961). Cellular senescence (or merely senescence) is a stable cellular response characterized by imposing a permanent and essentially irreversible cell cycle arrest and distinctive cellular phenotypic alterations induced by various stressors after a finite number of divisions, and it acts as a potentially significant contributor to aging and other age-related diseases (Childs et al., 2015), and otherwise has beneficial effects in restraining unnecessary cell proliferation like suppressing tumor cells (Calcinotto et al., 2019). Muñoz-Espín and Serrano (2014) concluded various stimuli and cellular contexts that produce cell senescence playing a key role in extensive physiological and pathological processes in 2014. From physiology to pathology, cellular senescence acts as a both beneficial and detrimental role, thus named a double-edged sword, but the underlying multiple molecular mechanisms of cellular senescence are similar. Cellular senescence was hypothesized as a process in response to different damaging stimuli that results in organismal aging, a detrimental role in physiology, and replicative senescence in cancer, a beneficial role in pathology (Childs et al., 2014). Considerable quantity of early work on the causes of cellular senescence was based on *in vitro* cell culture experiments whereby exposure to various types of oncogenic or metabolic stress, including radiation, reactive oxygen species (ROS), and DNA damage, and a cellular senescence growth arrest was successfully observed (Muñoz-Espín and Serrano, 2014)*.* Many of these plus other stressors such as oncogenic mutations, telomere erosion, epigenetic stress, and damage-associated molecular pattern proteins (DAMPs) have since been hypothesized as *in vivo* inducers of senescence (Kirkland and Tchkonia, 2017). Hernandez-Segura et al. (2018) described several main models of senescence induced by *in vitro* stimuli, classified in terms of triggers as well, including DNA damage-induced senescence, oncogene-induced senescence (OIS), oxidative stress-induced senescence, chemotherapy-induced senescence, mitochondrial dysfunction-induced senescence (MiDAS), epigenetically induced senescence and paracrine senescence. The intracellular signaling pathways that illustrate the mechanisms of cellular senescence are well understood *in vitro* while obscure *in vivo* (Childs et al., 2015). Opinions discussed above are accordant with the consensus reached by the International Cell Senescence Association (Gorgoulis et al., 2019). Cellular senescence is initiated by the damage sensor ataxia telangiectasia mutated (ATM)-p53-p21 axis, p16^INK4a^-Rb tumor suppressor networks and potentially other pathways (Tchkonia et al., 2013; Childs et al., 2015). Interestingly, the family of Retinoblastoma comprises three members—RB1, RB2/P130, and P107-that also play a role in regulating several aspects of cell life, including cell cycle, apoptosis, senescence, and differentiation. Accumulative senescent cells produce proinflammatory molecules, proteases and chemokines in aging tissues and organs, which is termed the senescence-associated secretory phenotype (SASP) (Tchkonia et al., 2013). In the developmental and physiological process, cellular senescence initiates a sequence of processes described as senescence-clearance-regeneration. During mammalian embryonic development, firstly in mouse embryos and followingly in human embryos, cellular senescence occurs in embryonic structures, which is substantiated by senescence-associated β-galactosidase (SA-β-GAL) assay and other molecular mechanisms, upregulations of cell cycle inhibitors (p15, p21 and p27) for example (Muñoz-Espín et al., 2013). Cellular senescence also takes place in physiological adult cells and organisms, especially in the proliferation of megakaryocytes, which is characterized by the increased SA-β-GAL activity and expression of p21 without p16, p53 or p27 (Besancenot et al., 2010). Diseases are universally acknowledged as disorders emerging with aging, in which cellular senescence plays a beneficial or detrimental role in the progressive procedures (Childs et al., 2015). That cellular senescence activates the p53 pathway was applied in the pro-senescent therapies for malignant tumors, the ultimate result turned out that tumor elimination followed. Also, it can be detectable that senescent surveillance helps tumor cell clearance (Kang et al., 2011). In contrast, cellular senescence aggravates the pathology in some diseases, such as Alzheimer’s disease, Parkinson’s disease, sarcopenia as well.

## 4 Mechanisms of Cellular Senescence in Sarcopenia

Aging causally contributes to cellular senescence, and meanwhile excessive senescent cells have been demonstrated to play a causal role in driving tissue aging, which may initiate the progression of chronic diseases (Song et al., 2020). Cellular senescence suppresses tumorigenesis *in vivo* by preventing potential cancer cells from proliferating, whilst in the dark side, senescent cells can disrupt local tissue integrity and contribute to onsets of several age-related pathologies, like sarcopenia (Campisi, 2001; Calcinotto et al., 2019). Sarcopenia is a geriatric syndrome in the musculoskeletal system, and by the year 2021, it has been recognized that there are multifactorial causes of age-related sarcopenia, such as impairments in neuromuscular function (Ryall et al., 2008) (loss of motor units innervating muscle), systemic inflammation (concluded as the SASP, characterized by higher level of cytokines), oxidative stress, decline in anabolic hormones (testosterone, growth hormone, insulin and insulin-like growth factors, and thyroid hormone) and mitochondrial dysfunction. However, the mechanisms involved in the development of sarcopenia are still poorly understood. To perform roles of senescent progress, senescent cells upregulate several senescent cell anti-apoptotic pathways—e.g., p53/p21^Cip1^/serpine pathways, p16^INK4a^/pRB pathways, as well as PI3K/Akt pathways, which interplay with each other and ultimately form networks (Kirkland et al., 2017)*.* Based on essential p53/p21^Cip1^/serpine pathways and p16^INK4a^/pRB pathways, herein we reviewed mechanisms of cellular senescence in sarcopenia including changes in muscle stem cell self-renewal function (Du et al., 2014) and the intracellular microenvironment of skeletal muscle. To this end, cellular senescence is associated with sarcopenia and aggravates the pathology (Mankhong et al., 2020), and considering the mechanisms of cellular senescence in sarcopenia, interventions on the signaling pathways are recommended, which may help to define the therapeutic strategies of new pharmacological discovery (can be named senolytic drugs). Multifaceted nature of cellular senescence has been rapidly evolving in recent years, but the understanding of the diverse biological functions of senescent cells remains far from complete and further research is still needed to elucidate how senescent cells influence the pathology of sarcopenia.

### 4.1 Pathways of Cellular Senescence

As mentioned above, cellular senescence was a phenomenon of cultured human diploid fibro-blasts first described by Leonard Hayflick and Paul Moorhead in 1960s (Hayflick and Moorhead, 1961). Subsequently, much work has been done to figure out the causes of cellular senescence and the following molecular mechanisms, which was based on *in vitro* cell culture experiments whereby extensive exposure to simultaneous, persistent and various types of stressors. Conclusions of cellular senescence relevant phenotypes have been drawn, which are elicited by telomere erosion (Harley et al., 1990), DNA damage (also known as DNA damage response, DDR), oncogenic mutations, epigenetic stress, reactive metabolites, proteotoxic stress, mitogens/IGF-1, DAMPs, cyclin-dependent kinase inhibitors (CDKi) (Hernandez-Segura et al., 2018) and so on. Diverse stimuli mentioned above can induce cellular senescence by converging on either or both of two major pathways, governed by two gatekeeper tumor suppressor proteins known as p53 and pRB, which initiate the senescence growth arrest. These pathways can interact with each other and halt the cell-cycle progression independently. The mechanisms that p53 and pRB signaling pathways regulate the progression of cellular senescence have been described by extensive experimental results. To some extent, the robust and novel markers of cellular senescence on the pathways governed by the gatekeeper proteins p53 and pRB may have a prognostic potential in delaying aging and treating cancers (Evangelou et al., 2017).

#### 4.1.1 The p53 Pathway

Cellular senescence is implemented by activation of the p53/p21^CIP1^ tumor suppressor pathway, a crucial pathway typically in response to persistent DNA damage (Herranz and Gil, 2018). DNA damage response (DDR), a signaling pathway in which protein kinases ataxia-telangiectasia mutated (ATM) and ATM-and Rad3-related (ATR) target the p53, which block cell-cycle progression, is triggered by the telomere erosion (Fagagna et al., 2003). Activated p53 in cellular senescence subsequently induce the transcription of the cyclin-dependent kinase inhibitor (CDKi) p21^CIP1^, which in turn blocks the activity of cyclin E-CDK2. The down-regulation expression of CDK2 initiate the hypophosphorylated Rb and cell cycle exit (di Fagagna, 2008). The DNA ARF locus activation inhibit the transcription of MDM2, which repress the protein p53 (Herranz and Gil, 2018).

Moiseeva et al. (2009) cultured normal human diploid IMR90 fibroblasts in Dulbecco’s modified Eagle’s medium, a oncogene-induced senescence, and draw the conclusion that mitochondrial dysfunction was an effector pathway to induce cellular senescence *in vitro* via p53 pathway.

Also, in both normal and cancer cells, Macip et al. showed that intracellular reactive oxygen species (ROS) increased the expression level of p53, correlated with the induction of cellular senescence. Reversely, cultured cells with ROS inhibitors ameliorated the p53-dependent cell fates. Moreover, the absence of p53 targets affecting mitochondrial function genes, Bax or PUMA, inhibited the ROS increase, presumably of mitochondrial origin (Macip et al., 2003). Telomeres progressively shorten throughout cell life, and the process is regulated by the activity of telomerase. Consequently, telomere shortening and telomerase inhibition induce the senescence, which can be experimentally demonstrated by up-regulation levels of cell cycle checkpoint genes p53/p21^CIP1^ (Shawi and Autexier, 2008; Shay, 2016). On the contrary, Campisi (2005) reviewed that experimental manipulations that induce the inactivation of p53 cause postmitotic senescent cells to resume growth, which demonstrated that the p53 signaling pathway in cellular senescence is reversible.

#### 4.1.2 The pRB Pathway

Cyclin-Dependent Kinase Inhibitors (CDKs) regulate multiple proteins relevant to the cell cycle progression, among which the CDKN2A (p16^INK4a^, hereafter p16) encode the CDKs, one main driver of cellular senescence. P16 is the significant mediator of the pRB signaling pathway. Contrary to p53/p21^CIP1^ signaling pathway, the p16^INK4a^/pRB signaling pathway activates an irreversible cellular senescence (Campisi, 2005). p16^INK4a^ blocks cell cycle progression by inhibiting the expression of CDK4/CDK6, which prevent the activation of pRB. p16^INK4a^/pRB pathway is regarded as a second barrier to proliferation, which can function either alone or in combination with the p53/p21^CIP1^ pathway (Childs et al., 2015). Activation of the INK4/ARF locus, which encodes three tumor suppressors: p16^INK4a^ and ARF (p14^ARF^ in human and p19^ARF^ in mouse), both encoded by the CDKN2A gene, and p15^INK4b^, encoded by CDKN2B, is the crucial senescence sensor in the p16^INK4a^/pRB pathway. p16^INK4^ and p15^INK4b^ inhibit the expression of CDK4 and CDK6, known as CDKIs, like p21^CIP1^, which arrest the cell cycle. INK4/ARF locus associates p16^INK4a^/pRB pathway with the p53/p21^CIP1^ pathway. ARF inhibits MDM2, which prevents expression of p53, thereby establishing a cross talk between p16^INK4a^/pRB pathway and p53/p21^CIP1^ pathway. Conversely, Harris et al. demonstrated that p53 regulates expression of ARF in p53^−/−^ mouse embryonic fibroblasts (Herranz and Gil, 2018; McHugh and Gil, 2018). Similar to p53/p21^CIP1^ pathway, p16^INK4a^/pRB pathway is activated primarily or secondarily in response to DDR (Campisi and d’Adda di Fagagna, 2007). Moreover, oncogenic RAS induces expression of p16^INK4a^ by up-regulating ETS and down-regulating Id proteins (Ohtani et al., 2001).

#### 4.1.3 Other Pathways

Aside from the major pathways regulating the cell cycle mentioned above, some other molecular pathways are demonstrated to participate in controlling cellular senescence. pRB is the protein product of the retinoblastoma gene (Rb), which plays a critical role in tumor suppression and regulating cell proliferation. Another two proteins, p107 and p130, together with pRB, are collectively called the “pocket proteins.” Studies with mice, whose “pocket proteins” selectively are knockout, showed transitions between proliferation and differentiation, as well as a shortening of the length of cell cycle, the single- double-, and triple-deficient cells exhibiting. The family of “pocket proteins” show overlapping functions, while an initial knockout analysis suggested that Rb/p107 and Rb/p130 played an ancillary role (Classon and Dyson, 2001). Three members of retinoblastoma gene family, namely RB1, RB2/P130, and P107, are associated with senescence, due to their role in controlling the cell cycle. Silencing experiments of each member of the family in mesenchymal stromal cells (MSCs) and fibroblasts from mouse and human tissues showed that RB1-P16 pathways in mouse cells are associated with senescence, whereas the RB2/P130-P27-P16 may regulate that process in human cells (Alessio et al., 2017).

### 4.2 Muscle Stem Cells Dysfunction

In general, adult skeletal muscle in mammals is a tissue staying stable under normal circumstances, but when injured, muscle stem cells (MuSCs, also known as satellite cells), play an indispensible role in the process of regenerating adult mammalian skeletal muscle (Yin et al., 2013). During the process of skeletal muscle regeneration, it is regulated by the dynamic interplay between intrinsic factors within satellite cells and extrinsic factors constituting the muscle stem cell niche/microenvironment. In detail, muscle stem cells reside in the quiescent G_0_ phase for prolonged period of time (Cheung and Rando, 2013), but when stimulated by damage or stresses, they are activated and enter the cell cycle, forming new fibres or self-renewing by proliferating (Yin et al., 2013). Cheung and Rando (2013) provided advances in deciphering the molecular mechanisms regulating adult stem cell (including MuSCs) quiescence, which provide potential avenues for understanding alterations of MuSCs in ageing.

Recent research has implicated cellular senescence-associated alterations in MuSCs as one of the causative mechanism of age-associated sarcopenia, which may result from loss of muscle regenerative potential. Skeletal muscle has plasticity of regeneration and remodeling due to MuSCs function, and the age-related decline of skeletal muscle mass and function (known as sarcopenia) is associated with loss of MuSCs regenerative capacity with aging (Sousa-Victor and Munoz-Canoves, 2016).Researches about some factors such as endothelin-1, TRIM32, and GSK-3α inducing MuSCs senescence, in which knockout of each of these factors results in muscle degeneration and sarcopenia development, have been carried out in this field (Kudryashova et al., 2012; Zhou et al., 2013; Alcalde-Estévez et al., 2020). Extrinsic changes and intrinsic changes negatively impact MuSC numbers and functionality, which is particularly pronounced in the sarcopenic muscle of both humans and mice (Sousa-Victor et al., 2014; Wan et al., 2021). Evidence in the past decade suggests the environment with aging contributes to MuSCs aging phenotype. Supportively, exposure of old satellite cells to young environment rejuvenates MuSCs and restore part of satellite cell function (Brack et al., 2007). Inversely, sharp regenerative decline of geriatric muscle stem cells was observed in geriatric mice (28–32 months in age) or 12-month-old SAMP8 mice (a mouse model of progeria), which is not attributed to a reduced satellite cell supply but regeneration efficiency (some adverse satellite-cell-intrinsic alterations), and such degeneration cannot be rejuvenated by a young host environment. This phenomenon is contrary to the result that extrinsic factors like a youthful environment can impact on intrinsic generative ability of MuSCs (Garcia-Prat et al., 2013).

Mechanistically, it has been confirmed that adult or even old satellite cells preserve their reversible quiescent state via repression of p16^INK4a^ in skeletal muscle homeostatic conditions. However, in geriatric mice, geriatric muscle stem cells switch reversible normal quiescent state into irreversible senescent state, in which MuSCs lose their intrinsic regenerative and self-renewal capacities, and the p16^INK4a^ (also called CDKN2a) silencing experimental results show that repression of p16^INK4a^ is capable of rejuvenating sarcopenic muscles (Sousa-Victor et al., 2014). The master regulator of senescence named p16^INK4a^ (CDKN2a) (Jacobs and de Lange, 2004) is the only significantly upregulated senescence gene in cluster G1, which was confirmed by the reduce of H2Aub mark in the INK4a locus (Sousa-Victor et al., 2014). p16^INK4a^ silencing through the delivery of short hairbin RNA (shRNA) targeting p16^INK4a^ (p16^INK4a^ RNA) into quiescent geriatric satellite cells restores satellite cell quiescence. Baker et al. (2011) designed a novel transgene, named INK-ATTAC, by making use of a biomarker for senescence, p16^Ink4a^, to target the symbolic senescent protein p16^Ink4a^. Additionally, application of INK-ATTAC in the BubR1 progeroid mouse induced elimination of p16^Ink4a^-positive senescent cells in skeletal muscle. Remarkably, treated mice (cohorts of BubR1^H/H^;INK-ATTAC-3 and -5 mice treated with AP20187 every third day beginning at 3 weeks of age) of both BubR1^H/H^;INK-ATTAC lines had substantially delayed onset of lordokyphosis (a measure of sarcopenia onset in this model) compared to untreated mice, which demonstrated that inactivation of p16^Ink4a^ in muscle stem cells in BubR1^H/H^ mice showed a marked decrease in senescence-associated β-galactosidase (SA-β-Gal) staining, a phenomenon of delaying sarcopenia. Active p16^INK4a^/Rb axis in human geriatric stem cells is the same as p16^INK4a^/Rb/E2F axis in murine satellite cells, which can induce stem-cell geroconversion. In aged MuSCs freshly purified through fluorescence activated cell sorting (FACS), hallmarks of aging-associated cell senescence such as the cell cycle inhibitors p16^Ink4a^ and p21^Cip1^ were elevated relevant to young MuSCs, shown by immunocytochemical analyses. Moreover, the matricellular protein CCN1 can upregulates expression of p16^INK4a^ and Rb (Du et al., 2014), and its upregulation is stimulated by Wnt-3a, which resides in the Wnt signaling pathway of MuSCs (Brack et al., 2007; Mankhong et al., 2020). Despite positive findings demonstrating expression of p16^Ink4a^ in senescent satellite cells, a recent study indicated that p16 ^Ink4a^- positive cells were not existed in skeletal muscle of elderly person (Yin et al., 2013; Idda et al., 2020).

Moreover, greater proportions of aged MuSCs (25%), relative to young (2%), exhibited active p38α/β mitogen-activated protein kinase (MAPK) signaling, by assaying stress signaling pathways through phosphoprotein flow cytometric analysis (Cosgrove et al., 2014). The p38α/β MAPK signaling pathway is depicted as a mechanism for the defectiveness of MuSCs, which may induce the sarcopenia by inducing p16^Ink4a^ expression and promoting cell senescence. Notably, alterations of FGF Receptor 1 and p38α/β MAPK (also known as MAPK14 and MAPK11 respectively) signaling in aged satellite cells cause a cell-autonomous loss in self-renewal, which underlies the mechanism of sarcopenia (Bernet et al., 2014). Bernet et al. collected RNA from FACS-isolated SCs, and quantitative analysis of myofiber-associated SCs revealed a 50% reduction in asymmetric phospho-p38 compared to young SCs. Notably, partial inhibition of p38αβ MAPK signaling with 25 μM SB203580 enhanced asymmetric phospho-p38 in aged SCs but not young SCs.

CCN1 sharply increases in skeletal muscle of aged experimental rats and mice, and it can upregulate expression of cell cycle arrest proteins and induce cellular senescence (Du et al., 2014). Du et al. treated cells with recombinant CCN1 and measured cell cycle arrest proteins in MPCs to identify the means by which CCN1 attenuates muscle cell proliferation, and found that p53 tumor inhibitor protein was increased 2.1-fold after 48 h of treatment with CCN1, p16^Ink4a^ was increased 2.7-fold, and hypophosphorylated form of RB (pRB) was 1.9-fold increased. Moreover, the CCN1-treated MPCs did express a senescence marker (SA-βgal). A recombinant adenovirus (Ad-wnt-3a) was used to mediate the overexpression of wnt-3a, and the results demonstrated that C2C12 myoblasts transduced with Ad-wnt-3a adenovirus exhibited up to a threefold increase in CCN1 protein. Normally, skeletal muscle regeneration is maintained by a Notch-p53 signaling axis, which can be illuminated that ligand-dependent stimulation of Notch activates p53 in MuSCs by inhibiting Mdm2 expression through Hey transcription factors pathway (Liu et al., 2018). However, the mechanism is impaired in aged animals, contributing to a decline in p53 associated with aging that exacerbates MuSC mitotic catastrophe, which serves as a dying means of MuSCs. Of note, mouse models with constitutively active p53 alleles imply that increased p53 activity results in ageing phenotypes (Tyner et al., 2002). Additionally, notch signaling regulates proliferation of MuSCs (Yin et al., 2013), so in old mice, inhibition of proliferation in satellite cells is shown by Smad-Notch signalling imbalance inducing the expression of p15^INK4b^ and p21^CIP1/WAF1^, two CDK inhibitor (Carlson et al., 2008; Sousa-Victor et al., 2014). The pathways mentioned above are complicated and molecules along the pathways may interact with those in the pathways of the senescence-associated secretory phenotype (abbreviated as SASP). SASP may account for loss of homeostasis in muscle and the defective regeneration of MuSCs may partly be down-regulated by the effects of SASP, but the relationship has not been investigated in this field.

Aside from a multitude of abnormal expressions of cell-cycle regulators, the functionality of epigenetic regulators driven by altered metabolism could also regulates DNA damage in MuSCs with aging. The signaling pathways in senescent MuSCs interacts with metabolic imbalance of mitochondrial epigenetic cofactors. Specifically, epigenome changes in ageing stem cells are caused by altered production of mitochondrial epigenetic cofactors, which is partly resulted from accumulation of mitochondrial DNA mutations. Levels of mitochondrial NAD^+^, known as an essential cofactor of the NAD-dependent protein deacetylase sirtuin 1 (SIRT1), are observed declining in aging stem cells. Zhang et al. demonstrates NAD^+^ impacts mitochondrial activity, regarding as a pivotal switch to modulate MuSCs senescence. Active SIRT1 promotes self-renewal of MuSCs and elevating levels of NAD^+^ improve the functionality of aged MuSCs in a SIRT1-dependent manner (Ryall et al., 2015; Zhang et al., 2016).

**FIGURE 1:**
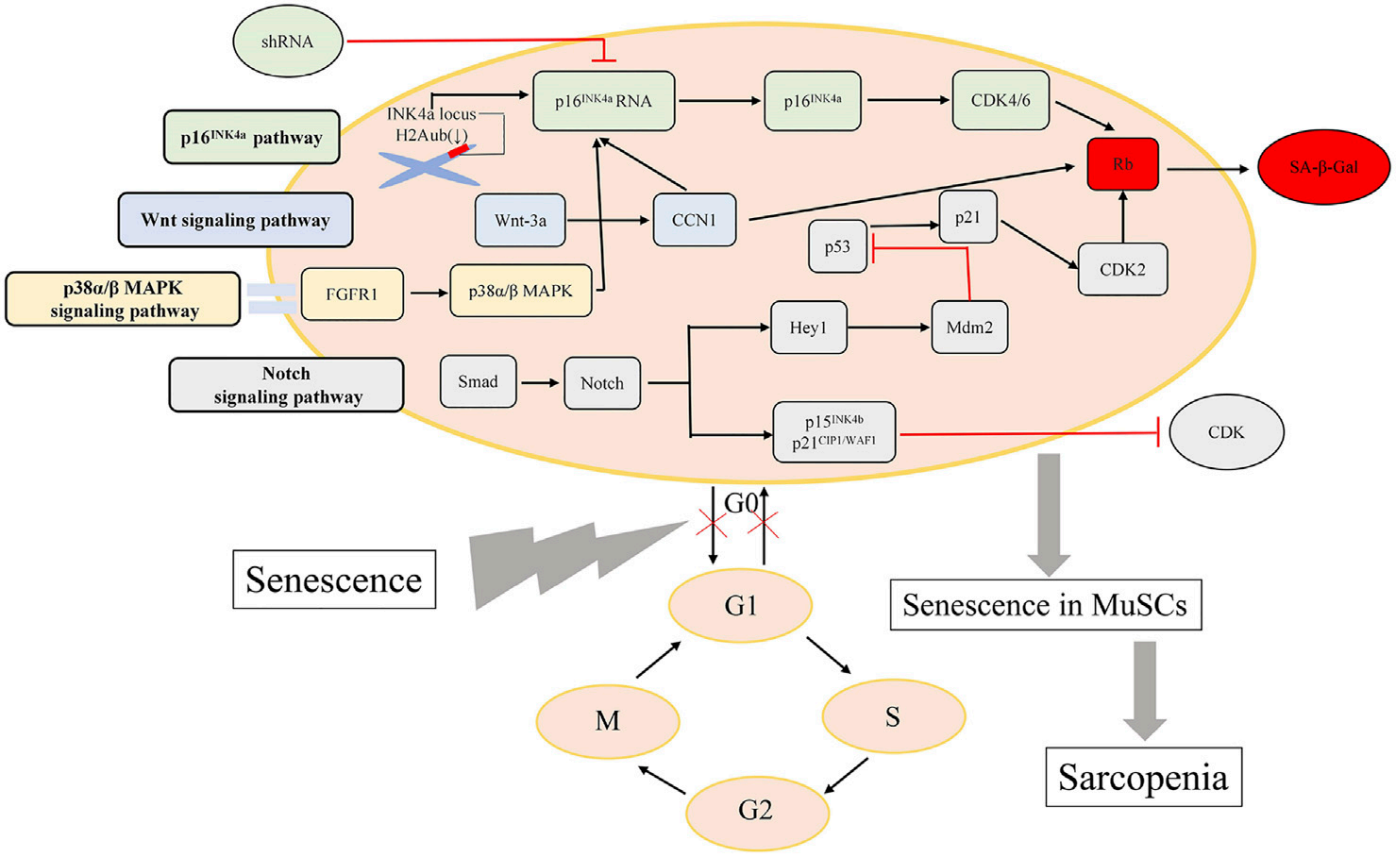
Major pathways regulating MuSCs dysfunction. The vital signaling pathways influencing the capacity of MuSCs regeneration are presented here.

The signaling pathways underlying the mechanisms of MuSCs dysfunction in sarcopenia mentioned above are depicted in Figure 1.

### 4.3 Senescence-Associated Secretory Phenotype

Cellular senescence has been implicated in chronic low-grade sterile inflammation, also known as inflammaging in sarcopenia, a hallmark of the ageing process, whereby the senescence-associated secretory phenotype (SASP), which transforms the microenvironment and impinges on the surrounding cells. SASP is characterized by the phenomenon that senescent cells secrete various inflammatory cytokines, chemokines, matrix remodeling proteases and growth factors. These components secreted by accumulative senescent cells in various tissues and organs are hypothesized to disrupt tissue structure and function (Baker et al., 2011). Accumulating data suggest that age-related inflammation in skeletal muscle is closely associated with age-related diseases including sarcopenia (Kalinkovich and Livshits, 2017) and the altered activation of the cell signaling pathway which promotes the inflammatory state further leads to one of the pathogenetic bases that underlie sarcopenia. Moreover, regulation of the SASP can be considered as novel therapeutic strategies in cellular senescence-associated diseases, especially sarcopenia (Guo, 2017).

Complicated pathways appear to contribute to the gene expression that underlie the SASP. Previous studies indicated that DNA damage response (DDR) is an essential stimulus for the SASP always through the p53 pathway. However, Freund et al. (2011) delineated that p38MAPK/NF-κB pathway is a crucial SASP regulatory pathway. Experimental results showed that the kinetics of p38MAPK activation closely paralleled the kinetics with which the SASP develops. Inhibition of p38MAPK activation by the small molecule SB203580 (SB) contributed to decreased levels of SASP secretions, which indicated that p38MAPK activity is sufficient to induce an SASP by promoting SASP component mRNA levels. Subsequently, p38MAPK acts primarily through NF-κB to induce the SASP. Nonetheless, p38MAPK regulates the SASP independently of the DDR, which is able to initiate the p53 pathway, one restraining p38MAPK conversely. Additionally, IL-1α signaling is an upstream regulator of senescence-associated IL-6/IL-8 secretion through both NF-κB and C/EBPβ pathways (Orjalo et al., 2009; Freund et al., 2010). Also, miR-146a/b acts as a negative regulator of inflammatory pathways by negatively regulating NF-κB activity (Bhaumik et al., 2009). NF-κB signaling pathway has also been implicated in regulating the atrophy of skeletal muscle and mechanically, myoblasts NF-κB is essential for TNF-α to mediate an inhibition of muscle differentiation in cultured C2C12 (Guttridge et al., 2000).

A wide range of SASP proteins includes IL-6, IL-8, IL-1, TNF-α, matrix metalloproteinases (MMPs, especially MMP-1, MMP-3), monocyte chemotactic proteins (MCPs, especially MCP-1,MCP-2, MCP-3), IGF binding proteins (IGFBPs, especially IGFBP-2, IGFBP-7), plasminogen activator inhibitor-1, granulocyte macrophage colony-stimulating factor (GM-CSF), growth regulated oncogene (GRO)α (Freund et al., 2010; Tchkonia et al., 2013)and C-reactive protein (CRP) (Papadopoulou, 2020). Subsequently, it is proposed that cytokines such as TNF-α, IL-6 (negatively regulated by anti-inflammatory cytokines, IL-10 for example (Curcio et al., 2016), IL-1 and CRP are contributing factors to a predisposition to sarcopenia by triggering the ubiquitin–protease system (Mitch and Goldberg, 1996). These factors act in an autocrine feedback loop to reinforce the senescence growth arrest as well as signal the immune system to clear senescent cells (Acosta et al., 2008; Freund et al., 2010; Tchkonia et al., 2013). Visser et al. (2002) observed that numerous studies in humans and animals showing higher IL-6 levels and higher TNF-α levels accompanying with advanced aging may predispose to lower muscle mass and strength, also called sarcopenia. One mechanism is that increased concentration of TNF-α induces activation of apoptosis in muscle cells. Also, higher levels of TNF-α and IL-1 are capable of blocking the differentiation of myoblasts (Cho et al., 2020). Additionally, TNF-α, as a major pro-inflammatory cytokine, was demonstrated a concomitant increase in an investigation, which implicated its function as a modifier of up-regulating MuRF1 and atrogin-1 expression, indicating a possible role of UbP-dependent atrophy during advanced age in aged muscle (Jo et al., 2012). One SASP component, TGFβ, triggers neighbouring cells entering senescence through a mechanism that generates ROS and DNA damage (Nelson et al., 2012). SASP components could be categorized into four classes: extracellular matrix/cytoskeleton/cell junctions; metabolic processes; ox-redox factors; and regulators of gene expression. It is speculated that the presence of some proteins that are exclusively expressed in all the analyzed senescent phenotypes may regulate key circuits for paracrine activity of senescent cells through the following paths: MMP2 - TIMP2; IGFBP3 – PAI-1 (SERPINE1); and Peroxiredoxin 6 – PARK7 – ERP46 – Major vault protein – Cathepsin D (Ozcan et al., 2016).

SASP induces chronic inflammation with aging, which can disrupt stem cell function because proteases secreted by senescent cells and the destructive activities of immune cells signaled by SASP components, can destroy stem cell niches, for example, by thickening the basal lamina around muscle satellite cells through extracellular matrix deposition, impeding satellite cell function (Gopinath and Rando, 2008). Such phenomenon is confirmed by a decrease in the number of SC resulting from the inflammatory process (Pascual-Fernández et al., 2020). And this may be a mechanism of age-related sarcopenia. Inflammation, mediated by increased secretion of proinflammatory and inflammatory cytokines, causes accelerated protein degradation (Fukushima et al., 2019), correlating with muscle fiber loss (Lima et al., 2020). Generally, senescence creates an inflammatory microenvironment by powerful paracrine activity that may lead to the elimination of senescent cells through signaling the immune system, which may result in sarcopenia.

Metabolic dysfunction is linked to aging at the organismal and molecular level according to several lines of studies. Specifically, in the molecular level, the pathway of mTOR fine-tunes metabolic regulation. A study could elucidate this by applying rapamycin, an inhibitor of the mTOR pathway, to genetically heterogeneous mice (Harrison et al., 2009). The mTOR pathway integrates key cellular processes to control metabolism, and its dysregulation has been implicated in metabolic disorders, as well as autophagy. In this regard, SASP is partly regulated by mTOR, and senescent growth arrest may be associated with SASP and mTOR. Still, more original studies should be done to further emphasize the intricate connection between metabolic stress and senescence in aging (Mankhong et al., 2020).

**FIGURE 2:**
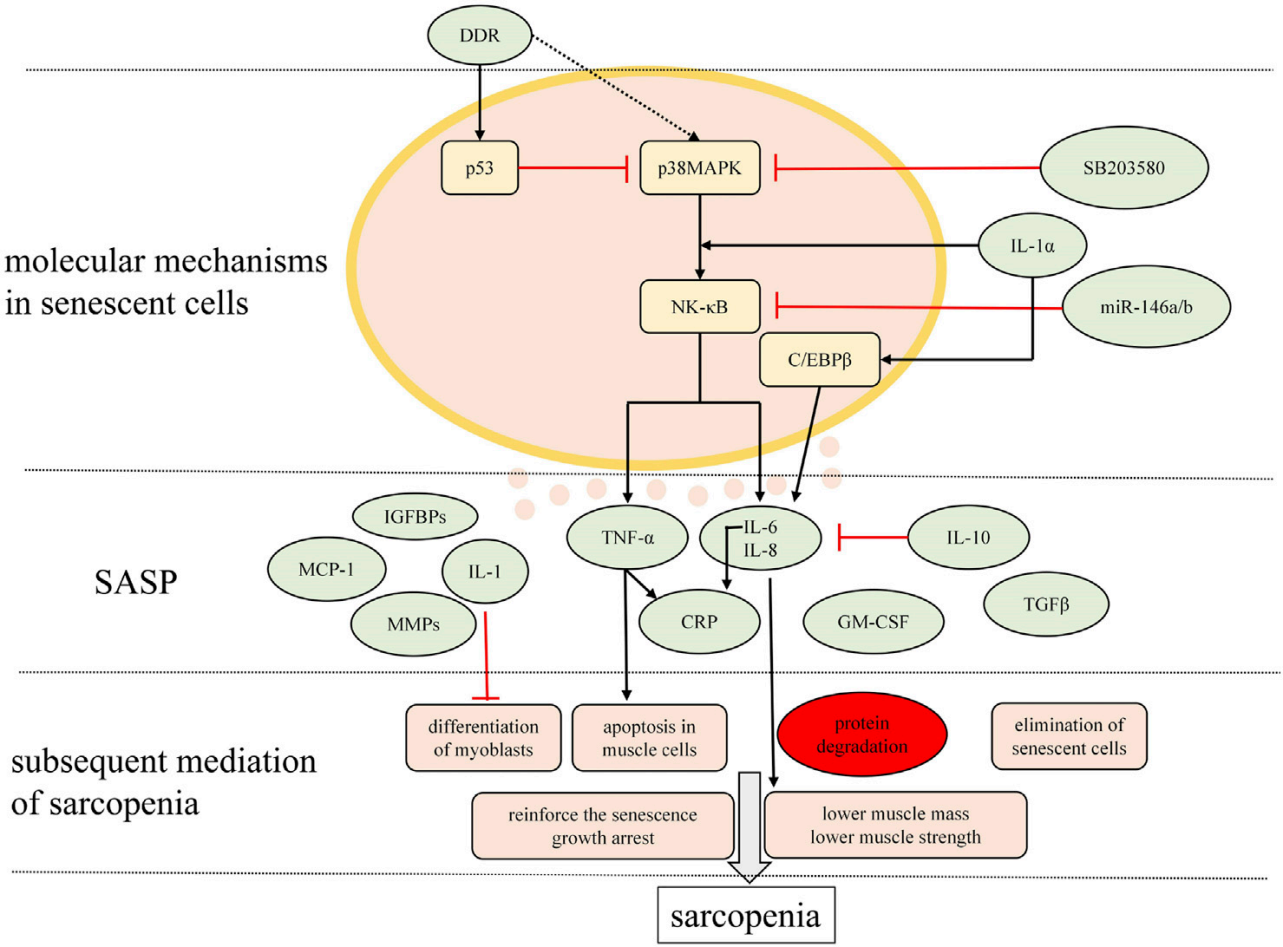
Major signaling pathways modulating the SASP and the main charactersitics of SASP relevant to sarcopenia. Mutiple molecules are secreted by senescent cells in musculosketal microenvironmet that possibly correlate with inflammaging in sarcopenia are presented here.

The signaling pathways underlying the mechanisms of the SASP in sarcopenia mentioned above are depicted in Figure 2.

## 5 Interventions Targeting Cellular Senescence Relevant to Sarcopenia

Cellular senescence is involved in various biological functions which play roles in promoting diseases such as cancer and other age-related disorders, which makes scientists regard cellular senescence as a therapeutic target for those diseases (Hernandez-Segura et al., 2018). New findings suggest that it may be feasible to delay sarcopenia by modulating fundamental aging mechanisms, such as cellular senescence. Collectively, existing aged preclinical models provide strong rationale for identifying and optimizing therapeutic strategies mediating cellular senescence to attenuate multiple chronic age-related diseases (Farr and Khosla, 2019). Cellular senescence itself and the following consequences it produces such as senescent cells accumulation and the SASP-associated chronic inflammation may be the desirable targets of interventions mitigating age-related sarcopenia. Therefore, interventions treating sarcopenia in aspect of cellular senescence could be categorized into three aspects: Interfering with pathways contributing to senescence-associated growth arrest, eliminating senescent cells, and interfering with the adverse effects of the SASP secreted by senescent cells. In contrast, numerous efforts have been made to develop effective new interventions irrespective of cellular senescence but concentrate on physical levels such as exercise regimens and hormone administration. Generally, rejuvenating the aged muscle with antisenescent therapies may be ideal (Muñoz-Espín and Serrano, 2014) though some adverse outcomes will accompany.

Interventions targeting cellular senescence may be potential pharmacological agents functioning through inhibition of cellular senescence. In this regard, pharmacological inhibition of the p38 MAPK pathway by SB203580 (Tocris) or SB202190 (EMD Chemicals) ameliorates age-associated sarcopenia and reveals a potential therapy for the treatment of progressive muscle wasting (Bernet et al., 2014; Cosgrove et al., 2014). McHugh et al. reviewed that treatment of mice with a Forkhead box protein O4 (FOXO4) inhibitor peptide can delay different aging phenotypes including p21^CIP1^ expression falling markedly in senescent cells, as localization of p53 to the nucleus by FOXO4 protects against p53 engaging the p53-mitochondrial signaling axis and apoptosis therein (Baar et al., 2017; McHugh and Gil, 2018). Properly upregulation of p53 lend the hope that eliminate deleterious aspects of cellular senescence without reversing the tumour-suppressive growth arrest to possibility. García-Cao et al. minimally increased the gene dosage of p53 in mice, from the normal dosage of two copies to just three copies, by introducing large genomic DNA segments containing the entire p53 gene, and such mice were called “Super p53” mice. And the result showed that ‘super p53’ mice had an enhanced response to DNA damage, were tumor resistant but aged normally (García-Cao et al., 2002). Although strategies interfering with pathways that lead to senescence-associated growth arrest are promising as mentioned above, they could be problematic if the mechanisms through which cellular senescence defends against cancer are compromised. As mentioned above, senescence in MuSCs contributes to sarcopenia. Inhibition of the JAK/STAT3 signaling pathway in MuSCs could be a target for improving the function of aged satellite cells, and ultimately attenuates sarcopenia (Sousa-Victor et al., 2015; Xie et al., 2021).

Strategies of specific elimination of senescent cells are highlighted for delaying sarcopenia. Senolytics induce cell death specifically by targeting pathways that lead to apoptosis. ABT263 (navitoclax), a potent inhibitor of BCL-2 and BCL-xL anti-apoptotic proteins, can be used to activate apoptosis of senescent cells (Prieto et al., 2020). Baker et al. (2011) showed that in BubR1 progeroid mouse, application of a novel transgene named INK-ATTAC targeting p16^Ink4a^ induced elimination of p16^Ink4a^-positive senescent cells upon administration of a drug, and the results demonstrated that life-long removal of p16^Ink4a^-expressing cells delayed onset of sarcopenia and even late-life clearance of those cells attenuated progression of already established sarcopenia. More importantly, clearing senescent cells was enhanced in progeroid mice but anticarcinogenic pathways upstream of senescence were not compromised. Additionally, Tchkonia et al. (2013) reviewed another two approaches possibly envisaged for removing senescent cells: 1) Specific antibodies targeting senescent cells, which involve biologicals recognizing epitopes that more highly express on senescent cells versus nonsenescent cells; 2) small molecules developed to selectively kill senescent cells.

The SASP is regarded as driver of age-related sarcopenia, so the inhibition of any the SASP regulators may lead to successful therapeutic interventions (Freund et al., 2010). Rapamycin, an inhibitor of mTOR, inhibits the SASP whereby a Nrf2-independent mechanism (Wang et al., 2017). In addition, general autophagy inhibition may serve as another way that delays cellular senescence and the SASP. Through the TOR-autophagy spatial coupling compartment (TASCC), autophagy generates a high flux of recycled amino acids and other metabolites, which are subsequently used by MTORC1 for supporting the massive synthesis of the SASP factors. Moreover, a key regulator of the SASP, GATA4, whose degradation can be mediated by autophagic receptor protein SQSTM1/p62, would accumulate by escaping from autophagic inhibition through decreased interaction between GATA4 and SQSTM1, and eventually initiate the SASP by activating NF-κB (Kang and Elledge, 2016). Therefore, it can be hypothesized that interventions inhibiting general autophagy that occurs in advanced ageing by targeting GATA4 may be potential for sarcopenia. Moreover, small-molecule approaches may be feasible to ameliorate the SASP. Glucocorticoid corticosterone and the related glucocorticoid cortisol suppress the SASP by selectively decreasing secretion of SASP components including several pro-inflammatory cytokines such as IL-6, IL-8 and suppressing IL-1α signaling by inhibiting NF-κB DNA binding and transactivation activity. And importantly, the glucocorticoids suppressed the SASP without reverting the tumor suppressive growth arrest, a major concern when strategies are targeted to inhibit the SASP (Laberge et al., 2012).

It has been demonstrated that physical inactivity is linked to loss of skeletal muscle strength and mass. Accumulating evidence suggests that exercise regimens as drugs including endurance training (ET) and resistance training (RT) can act on IGF-1/Akt/mTOR axis signaling pathways to delay age-related sarcopenia by increasing capacity of skeletal muscle to synthesize proteins when physical exercise programs are conducted in groups (Dhillon and Hasni, 2017; Nascimento et al., 2019). Additionally, physical exercise plays a role in modulating MuSC activity to delay age-related sarcopenia by mechanically increasing the expression of the neuronal form of NOS (nNOS) in the muscle and decreasing the expression of myostatin in muscle fibers (Pascual-Fernández et al., 2020). In animal models (mice model), exercise conditioning increased satellite cell number and activation status, as well as their capacity to differentiate into structurally and functionally appropriate myotubes, which rescue age-related loss of skeletal muscle mass (Snijders and Parise, 2017). The increase in the skeletal muscle after a period of resistance training has been quantified and certain research demonstrated the mechanisms of MuSC activation induced by RT an increase in mRNA of two cell cycle markers: cyclin-D1 and p21 (Kadi et al., 2004). The MuSC proliferation is associated with telomere length decreasing throughout life, but it has been manifested that moderate levels of physical activity (PA) have a benefit for skeletal muscle telomere length (Ludlow et al., 2008). Collectively, aerobic exercise (AT), resistance training (RT), and combination training (CT) interventions can reduce the dyskinesia of sarcopenia (Xie et al., 2020a). A randomized controlled trial (RCT) that older adults with sarcopenic obesity engaging in demonstrated muscle strength performance was better than the control group (Chen et al., 2017).

Sarcopenia appears accompanied by disorders of hormone levels, which indicates that hormone administration is possibly a potential therapy for sarcopenia (Papadopoulou, 2020). Anabolic hormones such as growth hormone, testosterone as well as selective androgen receptor modulators (SARMs) are predicted to improve lean body mass (LBM) but further evidence with a higher level of certainty of reversing sarcopenia is needed (Dent et al., 2018; Papadopoulou, 2020). MK-0773 (a SARM) supplementation for over 6 months in one quality RCT implemented in sarcopenic older female participant showed that skeletal muscle mass increased only.

The potential therapeutic implications and the targeted molecules, specific references as well are depicted in Table 1.

**TABLE 1:** Therapeutic interventions based on specific molecules on the signaling pathway of cellular senescence accounting for sarcopenia.

Interventions	Mechanisms	References
SB203580/SB202190	p38 MAPK	Brack et al. (2007), Idda et al. (2020)
FOXO4 inhibitor peptide	p21^CIP1^	Campisi (2005)
	p53	Lima et al. (2020)
Super p53	p53	Yin et al. (2013)
ABT263	BCL-2/BCL-xL	Baar et al. (2017)
INK-ATTAC	p16Ink4a	Sousa-Victor et al. (2014)
Rapamycin	mTOR (inhibit SASP)	García-Cao et al. (2002)
Autophagy inhibition	MTORC1/GATA4	Besancenot et al. (2010)
Glucocorticoid corticosterone	IL-6/IL-8/IL-1α signaling	Wang et al. (2017)
Exercise (ET/RT)	IGF-1/Akt/mTOR axis	Laberge et al. (2012), Kang and Elledge (2016)
	Cyclin-D1/p21/nNOS (MuSC)	Nelson et al. (2012), Nascimento et al. (2019)
	Telomere length	Snijders and Parise (2017)
GH/testosterone/SARMs (MK-0773)	Protein synthesis	Kadi et al. (2004), Xie et al. (2020a)
Inhibition of the JAK/STAT3	JAK/STAT3	Sousa-Victor et al. (2015)

## 6 Summary, Perspectives, and Future Directions

Sarcopenia is a primary geriatric disorder during the aging process, generally defined as a generalised and progressive skeletal muscle disorder resulting from aging as well as other pathologies. It involves a deterioration in muscle quantity and quality (diagnosis criterion), muscle strength (a key characteristic of sarcopenia) and muscle function, which contributes to various adverse physical performances such as falls, fall-related injury, slow movement, frailty and mortality. Nevertheless, underlying molecular mechanisms related to sarcopenia are not completely understood. Nonetheless, cellular senescence residing in the aging skeletal muscle environment can partly elucidate the mechanisms of sarcopenia. Multiple *in vitro* and *in vivo* experimental models (Mankhong et al., 2020) have been established to define the molecular and cellular changes as skeletal muscle ages, and herein, we review fundamental cellular senescence mechanisms leading to sarcopenia and provide potential interventions for prevention, delaying and treatment of sarcopenia as well as approaches for further development of anti-sarcopenia therapeutics.

p16^INK4a^/pRB pathway and p53/p21 pathway are widely acknowledged cell cycle inhibitor pathways triggering cellular senescence (Paez-Ribes et al., 2019). Senescence of the muscle stem cells is implicated as the underlying cause of loss of muscle regenerative potential, which ultimately induces ageing-associated sarcopenia. Similarly, the SASP in the senescent microenvironment bridges between cellular senescence and sarcopenia.

Interventions targeting cellular senescence and the SASP in sarcopenia may cause potential complications. For the SASP relevant interventions for sarcopenia, any inhibitions of SASP regulators such as p38MAPK inhibition may suppress p53 and p16 pathways, which may prevent beneficial effects of cellular senescence on other pathologies like suppressing cancer. Likewise, the mediators on the pathways regulating the SASP are involved in other cellular processes, so interventions targeting SASP regulation network possibly compromise other systems. We can draw the conclusion that further research is needed to identify more specific pathways or combinations of pathways underlying SASP.

Significant sarcopenia-related researches to be addressed should include increasing our understanding of molecular and cellular mechanisms underlying the pathophysiology of sarcopenia, the design of effective strategies to prevent and treat sarcopenia across the life course and carrying out some clinical trials based on interventions targeting cellular senescence in sarcopenia.

## References

[B1] Acosta J. C. O’Loghlen A. Banito A. Guijarro M. V. Augert A. Raguz S. (2008). Chemokine Signaling via the CXCR2 Receptor Reinforces Senescence. Cell 133 (6), 1006–1018. 10.1016/j.cell.2008.03.038 18555777

[B2] Alcalde-Estévez E. Asenjo-Bueno A. Sosa P. Olmos G. Plaza P. Caballero-Mora M. Á. (2020). Endothelin-1 Induces Cellular Senescence and Fibrosis in Cultured Myoblasts. A Potential Mechanism of Aging-Related Sarcopenia. Aging 12 (12), 11200–11223. 10.18632/aging.103450 32572011 PMC7343454

[B3] Alessio N. Capasso S. Ferone A. Di Bernardo G. Cipollaro M. Casale F. (2017). Misidentified Human Gene Functions with Mouse Models: The Case of the Retinoblastoma Gene Family in Senescence. Neoplasia 19 (10), 781–790. 10.1016/j.neo.2017.06.005 28865301 PMC5577395

[B4] Baar M. P. Brandt R. M. C. Putavet D. A. Klein J. D. D. Derks K. W. J. Bourgeois B. R. M. (2017). Targeted Apoptosis of Senescent Cells Restores Tissue Homeostasis in Response to Chemotoxicity and Aging. Cell 169 (1), 132–147.e16. 10.1016/j.cell.2017.02.031 28340339 PMC5556182

[B5] Baker D. J. Wijshake T. Tchkonia T. LeBrasseur N. K. Childs B. G. van de Sluis B. (2011). Clearance of p16Ink4a-Positive Senescent Cells Delays Ageing-Associated Disorders. Nature 479 (7372), 232–236. 10.1038/nature10600 22048312 PMC3468323

[B6] Bauer J. Morley J. E. Schols A. M. W. J. Ferrucci L. Cruz‐Jentoft A. J. Dent E. (2019). Sarcopenia: A Time for Action. An SCWD Position Paper. J. Cachexia Sarcopenia Muscle 10 (5), 956–961. 10.1002/jcsm.12483 31523937 PMC6818450

[B7] Bernet J. D. Doles J. D. Hall J. K. Kelly Tanaka K. Carter T. A. Olwin B. B. (2014). p38 MAPK Signaling Underlies a Cell-Autonomous Loss of Stem Cell Self-Renewal in Skeletal Muscle of Aged Mice. Nat. Med. 20 (3), 265–271. 10.1038/nm.3465 24531379 PMC4070883

[B8] Besancenot R. Chaligné R. Tonetti C. Pasquier F. Marty C. Lécluse Y. (2010). A Senescence-like Cell-Cycle Arrest Occurs during Megakaryocytic Maturation: Implications for Physiological and Pathological Megakaryocytic Proliferation. PLoS Biol. 8 (9), e1000476. 10.1371/journal.pbio.1000476 20838657 PMC2935456

[B9] Bhaumik D. Scott G. K. Schokrpur S. Patil C. K. Orjalo A. V. Rodier F. (2009). MicroRNAs miR-146a/b Negatively Modulate the Senescence-Associated Inflammatory Mediators IL-6 and IL-8. Aging 1 (4), 402–411. 10.18632/aging.100042 20148189 PMC2818025

[B10] Boengler K. Kosiol M. Mayr M. Schulz R. Rohrbach S. (2017). Mitochondria and Ageing: Role in Heart, Skeletal Muscle and Adipose Tissue. J. Cachexia Sarcopenia Muscle 8 (3), 349–369. 10.1002/jcsm.12178 28432755 PMC5476857

[B11] Brack A. S. Conboy M. J. Roy S. Lee M. Kuo C. J. Keller C. (2007). Increased Wnt Signaling During Aging Alters Muscle Stem Cell Fate and Increases Fibrosis. Science 317 (5839), 807–810. 10.1126/science.1144090 17690295

[B12] Brook M. S. Wilkinson D. J. Phillips B. E. Perez-Schindler J. Philp A. Smith K. (2016). Skeletal Muscle Homeostasis and Plasticity in Youth and Ageing: Impact of Nutrition and Exercise. Acta Physiol. 216 (1), 15–41. 10.1111/apha.12532 PMC4843955 26010896

[B13] Calcinotto A. Kohli J. Zagato E. Pellegrini L. Demaria M. Alimonti A. (2019). Cellular Senescence: Aging, Cancer, and Injury. Physiol. Rev. 99 (2), 1047–1078. 10.1152/physrev.00020.2018 30648461

[B14] Campisi J. (2001). Cellular Senescence as a Tumor-Suppressor Mechanism. Trends Cell Biol. 11 (11), S27–S31. 10.1016/s0962-8924(01)02151-110.1016/s0962-8924(01)82148-6 11684439

[B15] Campisi J. d’Adda di Fagagna F. (2007). Cellular Senescence: When Bad Things Happen to Good Cells. Nat. Rev. Mol. Cell Biol. 8 (9), 729–740. 10.1038/nrm2233 17667954

[B16] Campisi J. (2005). Senescent Cells, Tumor Suppression, and Organismal Aging: Good Citizens, Bad Neighbors. Cell 120 (4), 513–522. 10.1016/j.cell.2005.02.003 15734683

[B17] Carlson M. E. Hsu M. Conboy I. M. (2008). Imbalance Between pSmad3 and Notch Induces CDK Inhibitors in Old Muscle Stem Cells. Nature 454 (7203), 528–532. 10.1038/nature07034 18552838 PMC7761661

[B18] Chen H.-T. Chung Y.-C. Chen Y.-J. Ho S.-Y. Wu H.-J. (2017). Effects of Different Types of Exercise on Body Composition, Muscle Strength, and IGF-1 in the Elderly with Sarcopenic Obesity. J. Am. Geriatr. Soc. 65 (4), 827–832. 10.1111/jgs.14722 28205203

[B19] Chen L.-K. Liu L.-K. Woo J. Assantachai P. Auyeung T.-W. Bahyah K. S. (2014). Sarcopenia in Asia: Consensus Report of the Asian Working Group for Sarcopenia. J. Am. Med. Dir. Assoc. 15 (2), 95–101. 10.1016/j.jamda.2013.11.025 24461239

[B20] Cheung T. H. Rando T. A. (2013). Molecular Regulation of Stem Cell Quiescence. Nat. Rev. Mol. Cell Biol. 14 (6), 329–340. 10.1038/nrm3591 23698583 PMC3808888

[B21] Childs B. G. Baker D. J. Kirkland J. L. Campisi J. Deursen J. M. (2014). Senescence and Apoptosis: Dueling or Complementary Cell Fates? EMBO Rep. 15 (11), 1139–1153. 10.15252/embr.201439245 25312810 PMC4253488

[B22] Childs B. G. Durik M. Baker D. J. van Deursen J. M. (2015). Cellular Senescence in Aging and Age-Related Disease: From Mechanisms to Therapy. Nat. Med. 21 (12), 1424–1435. 10.1038/nm.4000 26646499 PMC4748967

[B23] Cho J. Choi Y. Sajgalik P. No M.-H. Lee S.-H. Kim S. (2020). Exercise as a Therapeutic Strategy for Sarcopenia in Heart Failure: Insights into Underlying Mechanisms. Cells 9 (10), 2284. 10.3390/cells9102284 PMC7602002 33066240

[B24] Classon M. Dyson N. (2001). p107 and P130: Versatile Proteins with Interesting Pockets. Exp. Cell Res. 264 (1), 135–147. 10.1006/excr.2000.5135 11237530

[B25] Cosgrove B. D. Gilbert P. M. Porpiglia E. Mourkioti F. Lee S. P. Corbel S. Y. (2014). Rejuvenation of the Muscle Stem Cell Population Restores Strength to Injured Aged Muscles. Nat. Med. 20 (3), 255–264. 10.1038/nm.3464 24531378 PMC3949152

[B26] Cruz-Jentoft A. J. Baeyens J. P. Bauer J. M. Boirie Y. Cederholm T. Landi F. (2010a). Sarcopenia: European Consensus on Definition and Diagnosis: Report of the European Working Group on Sarcopenia in Older People. Age Ageing 39 (4), 412–423. 10.1093/ageing/afq034 20392703 PMC2886201

[B27] Cruz-Jentoft A. J. Bahat G. Bauer J. Boirie Y. Bruyère O. Cederholm T. (2019). Sarcopenia: Revised European Consensus on Definition and Diagnosis. Age Ageing 48 (1), 16–31. 10.1093/ageing/afy169 30312372 PMC6322506

[B28] Cruz-Jentoft A. J. Landi F. Topinková E. Michel J.-P. (2010b). Understanding Sarcopenia as a Geriatric Syndrome. Curr. Opin. Clin. Nutr. Metab. Care 13 (1), 1–7. 10.1097/MCO.0b013e328333c1c1 19915458

[B29] Curcio F. Ferro G. Basile C. Liguori I. Parrella P. Pirozzi F. (2016). Biomarkers in Sarcopenia: A Multifactorial Approach. Exp. Gerontol. 85, 1–8. 10.1016/j.exger.2016.09.007 27633530

[B30] Dennison E. M. Sayer A. A. Cooper C. (2017). Epidemiology of Sarcopenia and Insight Into Possible Therapeutic Targets. Nat. Rev. Rheumatol. 13 (6), 340–347. 10.1038/nrrheum.2017.60 28469267 PMC5444517

[B31] DentE.MorleyJ. E.Cruz-JentoftA. J.AraiH.KritchevskyS. B.GuralnikJ. (2018). International Clinical Practice Guidelines for Sarcopenia (ICFSR): Screening, Diagnosis and Management. J. Nutr. Health Aging 22 (10), 1148–1161. 10.1007/s12603-018-1139-9 30498820

[B32] Dhillon R. J. S. Hasni S. (2017). Pathogenesis and Management of Sarcopenia. Clin. Geriatr. Med. 33 (1), 17–26. 10.1016/j.cger.2016.08.002 27886695 PMC5127276

[B33] di Fagagna F. D. (2008). Living on a Break: Cellular Senescence as a DNA-Damage Response. Nat. Rev. Cancer 8 (7), 512–522. 10.1038/nrc2440 18574463

[B34] Du J. Klein J. D. Hassounah F. Zhang J. Zhang C. Wang X. H. (2014). Aging Increases CCN1 Expression Leading to Muscle Senescence. Am. J. Physiol. Cell Physiol. 306 (1), C28–C36. 10.1152/ajpcell.00066.2013 24196529 PMC3919975

[B35] Evangelou K. Lougiakis N. Rizou S. V. Kotsinas A. Kletsas D. Muñoz-Espín D. (2017). Robust, Universal Biomarker Assay to Detect Senescent Cells in Biological Specimens. Aging Cell 16 (1), 192–197. 10.1111/acel.12545 28165661 PMC5242262

[B36] Fagagna F. D. A. D. Reaper P. M. Clay-Farrace L. Fiegler H. Carr P. Von Zglinicki T. (2003). A DNA Damage Checkpoint Response in Telomere-Initiated Senescence. Nature 426 (6963), 194–198. 10.1038/nature02118 14608368

[B37] Farr J. N. Khosla S. (2019). Cellular Senescence in Bone. Bone 121, 121–133. 10.1016/j.bone.2019.01.015 30659978 PMC6485943

[B38] Freund A. Orjalo A. V. Desprez P.-Y. Campisi J. (2010). Inflammatory Networks During Cellular Senescence: Causes and Consequences. Trends Mol. Med. 16 (5), 238–246. 10.1016/j.molmed.2010.03.003 20444648 PMC2879478

[B39] Freund A. Patil C. K. Campisi J. (2011). p38MAPK Is a Novel DNA Damage Response-Independent Regulator of the Senescence-Associated Secretory Phenotype. EMBO J. 30 (8), 1536–1548. 10.1038/emboj.2011.69 21399611 PMC3102277

[B40] Fukuoka Y. Narita T. Fujita H. Morii T. Sato T. Sassa M. H. (2019). Importance of Physical Evaluation Using Skeletal Muscle Mass index and Body Fat Percentage to Prevent Sarcopenia in Elderly Japanese Diabetes Patients. J. Diabetes Investig. 10 (2), 322–330. 10.1111/jdi.12908 PMC6400206 30098231

[B41] Fukushima H. Fujii Y. Koga F. (2019). Metabolic and Molecular Basis of Sarcopenia: Implications in the Management of Urothelial Carcinoma. Int. J. Mol. Sci. 20 (3), 760. 10.3390/ijms20030760 PMC6387186 30754663

[B42] García-Cao I. García-Cao M. Martín-Caballero J. Criado L. M. Klatt P. Flores J. M. (2002). ‘Super p53’ Mice Exhibit Enhanced DNA Damage Response, are Tumor Resistant and Age Normally. EMBO J. 21 (22), 6225–6235. 10.1093/emboj/cdf595 12426394 PMC137187

[B43] García-Prat L. Sousa-Victor P. Muñoz-Cánoves P. (2013). Functional Dysregulation of Stem Cells during Aging: A Focus on Skeletal Muscle Stem Cells. FEBS J. 280 (17), 4051–4062. 10.1111/febs.12221 23452120

[B44] Gopinath S. D. Rando T. A. (2008). Stem Cell Review Series: Aging of the Skeletal Muscle Stem Cell Niche. Aging Cell 7 (4), 590–598. 10.1111/j.1474-9726.2008.00399.x 18462272

[B45] Gorgoulis V. Adams P. D. Alimonti A. Bennett D. C. Bischof O. Bishop C. (2019). Cellular Senescence: Defining a Path Forward. Cell 179 (4), 813–827. 10.1016/j.cell.2019.10.005 31675495

[B46] Guo M. (2017). Cellular Senescence and Liver Disease: Mechanisms and Therapeutic Strategies. Biomed. Pharmacother. 96, 1527–1537. 10.1016/j.biopha.2017.11.075 29174037

[B47] Guttridge D. C. Mayo M. W. Madrid L. V. Wang C.-Y. Baldwin Jr. A. S. Jr. (2000). NF-κB-Induced Loss of MyoD Messenger RNA: Possible Role in Muscle Decay and Cachexia. Science 289 (5488), 2363–2366. 10.1126/science.289.5488.2363 11009425

[B48] Harley C. B. Futcher A. B. Greider C. W. (1990). Telomeres Shorten during Ageing of Human Fibroblasts. Nature 345 (6274), 458–460. 10.1038/345458a0 2342578

[B49] Harrison D. E. Strong R. Sharp Z. D. Nelson J. F. Astle C. M. Flurkey K. (2009). Rapamycin Fed Late in Life Extends Lifespan in Genetically Heterogeneous Mice. Nature 460 (7253), 392–395. 10.1038/nature08221 19587680 PMC2786175

[B50] Hayflick L. Moorhead P. S. (1961). The Serial Cultivation of Human Diploid Cell Strains. Exp. Cell Res. 25, 585–621. 10.1016/0014-4827(61)90192-6 13905658

[B51] Hernandez-Segura A. Nehme J. Demaria M. (2018). Hallmarks of Cellular Senescence. Trends Cell Biol. 28 (6), 436–453. 10.1016/j.tcb.2018.02.001 29477613

[B52] Herranz N. Gil J. (2018). Mechanisms and Functions of Cellular Senescence. J. Clin. Invest. 128 (4), 1238–1246. 10.1172/jci95148 29608137 PMC5873888

[B53] Idda M. L. McClusky W. G. Lodde V. Munk R. Abdelmohsen K. Rossi M. (2020). Survey of Senescent Cell Markers with Age in Human Tissues. Aging 12 (5), 4052–4066. 10.18632/aging.102903 32160592 PMC7093180

[B54] Izumida T. Imamura T. (2020). Sarcopenia in Patients with Cardiovascular Disease. J. Cardiol. 76 (6), 636. 10.1016/j.jjcc.2020.06.013 32636126

[B55] Jacobs J. J. L. de Lange T. (2004). Significant Role for p16INK4a in p53-Independent Telomere-Directed Senescence. Curr. Biol. 14 (24), 2302–2308. 10.1016/j.cub.2004.12.025 15620660

[B56] Jo E. Lee S. R. Park B. S. Kim J. S. (2012). Potential Mechanisms Underlying the Role of Chronic Inflammation in Age-Related Muscle Wasting. Aging Clin. Exp. Res. 24 (5), 412–422. 10.3275/8464 22717404

[B57] Kadi F. Schjerling P. Andersen L. L. Charifi N. Madsen J. L. Christensen L. R. (2004). The Effects of Heavy Resistance Training and Detraining on Satellite Cells in Human Skeletal Muscles. J. Physiol. 558 (Pt 3), 1005–1012. 10.1113/jphysiol.2004.065904 15218062 PMC1665027

[B58] Kalinkovich A. Livshits G. (2017). Sarcopenic Obesity or Obese Sarcopenia: A Cross Talk between Age-Associated Adipose Tissue and Skeletal Muscle Inflammation as a Main Mechanism of the Pathogenesis. Ageing Res. Rev. 35, 200–221. 10.1016/j.arr.2016.09.008 27702700

[B59] Kang C. Elledge S. J. (2016). How Autophagy Both Activates and Inhibits Cellular Senescence. Autophagy 12 (5), 898–899. 10.1080/15548627.2015.1121361 27129029 PMC4854549

[B60] Kang T.-W. Yevsa T. Woller N. Hoenicke L. Wuestefeld T. Dauch D. (2011). Senescence Surveillance of Pre-Malignant Hepatocytes Limits Liver Cancer Development. Nature 479 (7374), 547–551. 10.1038/nature10599 22080947

[B61] Kirkland J. L. Tchkonia T. (2017). Cellular Senescence: A Translational Perspective. EBioMedicine 21, 21–28. 10.1016/j.ebiom.2017.04.013 28416161 PMC5514381

[B62] Kirkland J. L. Tchkonia T. Zhu Y. Niedernhofer L. J. Robbins P. D. (2017). The Clinical Potential of Senolytic Drugs. J. Am. Geriatr. Soc. 65 (10), 2297–2301. 10.1111/jgs.14969 28869295 PMC5641223

[B63] Kudryashova E. Kramerova I. Spencer M. J. (2012). Satellite Cell Senescence Underlies Myopathy in a Mouse Model of Limb-Girdle Muscular Dystrophy 2H. J. Clin. Invest. 122 (5), 1764–1776. 10.1172/jci59581 22505452 PMC3336976

[B64] Laberge R.-M. Zhou L. Sarantos M. R. Rodier F. Freund A. de Keizer P. L. J. (2012). Glucocorticoids Suppress Selected Components of the Senescence-Associated Secretory Phenotype. Aging Cell 11 (4), 569–578. 10.1111/j.1474-9726.2012.00818.x 22404905 PMC3387333

[B65] Lima D. P. de Almeida S. B. Bonfadini J. d. C. de Luna J. R. G. de Alencar M. S. Pinheiro-Neto E. B. (2020). Clinical Correlates of Sarcopenia and Falls in Parkinson’s Disease. PLoS One 15 (3), e0227238. 10.1371/journal.pone.0227238 32191713 PMC7082018

[B66] Liu L. Charville G. W. Cheung T. H. Yoo B. Santos P. J. Schroeder M. (2018). Impaired Notch Signaling Leads to a Decrease in p53 Activity and Mitotic Catastrophe in Aged Muscle Stem Cells. Cell Stem Cell 23 (4), 544–556.e4. 10.1016/j.stem.2018.08.019 30244867 PMC6173623

[B67] Ludlow A. T. Zimmerman J. B. Witkowski S. Hearn J. W. Hatfield B. D. Roth S. M. (2008). Relationship Between Physical Activity Level, Telomere Length, and Telomerase Activity. Med. Sci. Sports Exerc. 40 (10), 1764–1771. 10.1249/MSS.0b013e31817c92aa 18799986 PMC2581416

[B68] Macip S. Igarashi M. Berggren P. Yu J. Lee S. W. Aaronson S. A. (2003). Influence of Induced Reactive Oxygen Species in p53-Mediated Cell Fate Decisions. Mol. Cell Biol. 23 (23), 8576–8585. 10.1128/mcb.23.23.8576-8585.2003 14612402 PMC262651

[B69] Mankhong S. Kim S. Moon S. Kwak H.-B. Park D.-H. Kang J.-H. (2020). Experimental Models of Sarcopenia: Bridging Molecular Mechanism and Therapeutic Strategy. Cells 9 (6), 1385. 10.3390/cells9061385 PMC7348939 32498474

[B70] McHugh D. Gil J. (2018). Senescence and Aging: Causes, Consequences, and Therapeutic Avenues. J. Cell Biol. 217 (1), 65–77. 10.1083/jcb.201708092 29114066 PMC5748990

[B71] Mitch W. E. Goldberg A. L. (1996). Mechanisms of Muscle Wasting - The Role of the Ubiquitin-Proteasome Pathway. N. Engl. J. Med. 335 (25), 1897–1905. 10.1056/nejm199612193352507 8948566

[B72] Moiseeva O. Bourdeau V. Roux A. Deschênes-Simard X. Ferbeyre G. (2009). Mitochondrial Dysfunction Contributes to Oncogene-Induced Senescence. Mol. Cell Biol. 29 (16), 4495–4507. 10.1128/mcb.01868-08 19528227 PMC2725737

[B73] Muñoz-Espín D. Cañamero M. Maraver A. Gómez-López G. Contreras J. Murillo-Cuesta S. (2013). Programmed Cell Senescence during Mammalian Embryonic Development. Cell 155 (5), 1104–1118. 10.1016/j.cell.2013.10.019 24238962

[B74] Muñoz-Espín D. Serrano M. (2014). Cellular Senescence: from Physiology to Pathology. Nat. Rev. Mol. Cell Biol. 15 (7), 482–496. 10.1038/nrm3823 24954210

[B75] Nascimento C. M. Ingles M. Salvador-Pascual A. Cominetti M. R. Gomez-Cabrera M. C. Viña J. (2019). Sarcopenia, Frailty and Their Prevention by Exercise. Free Radic. Biol. Med. 132, 42–49. 10.1016/j.freeradbiomed.2018.08.035 30176345

[B76] Nelson G. Wordsworth J. Wang C. Jurk D. Lawless C. Martin‐Ruiz C. (2012). A Senescent Cell Bystander Effect: Senescence‐induced Senescence. Aging Cell 11 (2), 345–349. 10.1111/j.1474-9726.2012.00795.x 22321662 PMC3488292

[B77] Ohtani N. Zebedee Z. Huot T. J. G. Stinson J. A. Sugimoto M. Ohashi Y. (2001). Opposing Effects of Ets and Id Proteins on p16INK4a Expression during Cellular Senescence. Nature 409 (6823), 1067–1070. 10.1038/35059131 11234019

[B78] Orjalo A. V. Bhaumik D. Gengler B. K. Scott G. K. Campisi J. (2009). Cell Surface-Bound IL-1 Is an Upstream Regulator of the Senescence-Associated IL-6/IL-8 Cytokine Network. Proc. Natl. Acad. Sci. 106 (40), 17031–17036. 10.1073/pnas.0905299106 19805069 PMC2761322

[B79] Özcan S. Alessio N. Acar M. B. Mert E. Omerli F. Peluso G. (2016). Unbiased Analysis of Senescence Associated Secretory Phenotype (SASP) to Identify Common Components Following Different Genotoxic Stresses. Aging 8 (7), 1316–1329. 10.18632/aging.100971 27288264 PMC4993333

[B80] Paez‐Ribes M. González‐Gualda E. Doherty G. J. Muñoz‐Espín D. (2019). Targeting Senescent Cells in Translational Medicine. EMBO Mol. Med. 11 (12), e10234. 10.15252/emmm.201810234 31746100 PMC6895604

[B81] Papadopoulou S. K. (2020). Sarcopenia: A Contemporary Health Problem Among Older Adult Populations. Nutrients 12 (5), 1293. 10.3390/nu12051293 PMC7282252 32370051

[B82] Pascual-Fernández J. Fernández-Montero A. Córdova-Martínez A. Pastor D. Martínez-Rodríguez A. Roche E. (2020). Sarcopenia: Molecular Pathways and Potential Targets for Intervention. Ijms 21 (22), 8844. 10.3390/ijms21228844 PMC7700275 33266508

[B83] Prieto L. I. Graves S. I. Baker D. J. (2020). Insights from *In Vivo* Studies of Cellular Senescence. Cells 9 (4), 954. 10.3390/cells9040954 PMC7226957 32295081

[B84] Rong S. Wang L. Peng Z. Liao Y. Li D. Yang X. (2020). The Mechanisms and Treatments for Sarcopenia: Could Exosomes be a Perspective Research Strategy in the Future? J. Cachexia Sarcopenia Muscle 11 (2), 348–365. 10.1002/jcsm.12536 31989804 PMC7113536

[B85] Rosenberg I. H. (1997). Sarcopenia: Origins and Clinical Relevance. J. Nutr. 127 (5 Suppl. l), 990s–991s. 10.1093/jn/127.5.990S 9164280

[B86] Ryall J. G. Dell’Orso S. Derfoul A. Juan A. Zare H. Feng X. (2015). The NAD+-Dependent SIRT1 Deacetylase Translates a Metabolic Switch into Regulatory Epigenetics in Skeletal Muscle Stem Cells. Cell Stem Cell 16 (2), 171–183. 10.1016/j.stem.2014.12.004 25600643 PMC4320668

[B87] Ryall J. G. Schertzer J. D. Lynch G. S. (2008). Cellular and Molecular Mechanisms Underlying Age-Related Skeletal Muscle Wasting and Weakness. Biogerontology 9 (4), 213–228. 10.1007/s10522-008-9131-0 18299960

[B88] Shawi M. Autexier C. (2008). Telomerase, Senescence and Ageing. Mech. Ageing Develop. 129 (1-2), 3–10. 10.1016/j.mad.2007.11.007 18215413

[B89] Shay J. W. (2016). Role of Telomeres and Telomerase in Aging and Cancer. Cancer Discov. 6 (6), 584–593. 10.1158/2159-8290.Cd-16-0062 27029895 PMC4893918

[B90] Sierzega M. Chrzan R. (2019). Sarcopenia Associated with Gastric Cancer. J. Surg. Oncol. 120 (8), 1509. 10.1002/jso.25744 31646640

[B91] Snijders T. Parise G. (2017). Role of Muscle Stem Cells in Sarcopenia. Curr. Opin. Clin. Nutr. Metab. Care 20 (3), 186–190. 10.1097/MCO.0000000000000360 28376051

[B92] Song P. An J. Zou M.-H. (2020). Immune Clearance of Senescent Cells to Combat Ageing and Chronic Diseases. Cells 9 (3), 671. 10.3390/cells9030671 PMC7140645 32164335

[B93] Sousa-Victor P. García-Prat L. Serrano A. L. Perdiguero E. Muñoz-Cánoves P. (2015). Muscle Stem Cell Aging: Regulation and Rejuvenation. Trends Endocrinol. Metab. 26 (6), 287–296. 10.1016/j.tem.2015.03.006 25869211

[B94] Sousa-Victor P. Gutarra S. García-Prat L. Rodriguez-Ubreva J. Ortet L. Ruiz-Bonilla V. (2014). Geriatric Muscle Stem Cells Switch Reversible Quiescence into Senescence. Nature 506 (7488), 316–321. 10.1038/nature13013 24522534

[B95] Sousa-Victor P. Muñoz-Cánoves P. (2016). Regenerative Decline of Stem Cells in Sarcopenia. Mol. Aspects Med. 50, 109–117. 10.1016/j.mam.2016.02.002 26921790

[B96] Tchkonia T. Zhu Y. van Deursen J. Campisi J. Kirkland J. L. (2013). Cellular Senescence and the Senescent Secretory Phenotype: Therapeutic Opportunities. J. Clin. Invest. 123 (3), 966–972. 10.1172/jci64098 23454759 PMC3582125

[B97] Tyner S. D. Venkatachalam S. Choi J. Jones S. Ghebranious N. Igelmann H. (2002). p53 Mutant Mice that Display Early Ageing-Associated Phenotypes. Nature 415 (6867), 45–53. 10.1038/415045a 11780111

[B98] Visser M. Pahor M. Taaffe D. R. Goodpaster B. H. Simonsick E. M. Newman A. B. (2002). Relationship of Interleukin-6 and Tumor Necrosis Factor- With Muscle Mass and Muscle Strength in Elderly Men and Women: The Health ABC Study. J. Gerontol. A Biol. Sci. Med. Sci. 57 (5), M326–M332. 10.1093/gerona/57.5.m326 11983728

[B99] Wan M. Gray-Gaillard E. F. Elisseeff J. H. (2021). Cellular Senescence in Musculoskeletal Homeostasis, Diseases, and Regeneration. Bone Res. 9 (1), 41. 10.1038/s41413-021-00164-y 34508069 PMC8433460

[B100] Wang R. Yu Z. Sunchu B. Shoaf J. Dang I. Zhao S. (2017). Rapamycin Inhibits the Secretory Phenotype of Senescent Cells by a Nrf2-independent Mechanism. Aging Cell 16 (3), 564–574. 10.1111/acel.12587 28371119 PMC5418203

[B101] Wiedmer P. Jung T. Castro J. P. Pomatto L. C. D. Sun P. Y. Davies K. J. A. (2021). Sarcopenia - Molecular Mechanisms and Open Questions. Ageing Res. Rev. 65, 101200. 10.1016/j.arr.2020.101200 33130247

[B102] Xie W.-Q. Xiao G.-L. Hu P.-W. He Y.-Q. Lv S. Xiao W.-F. (2020a). Possible Sarcopenia: Early Screening and Intervention-Narrative Review. Ann. Palliat. Med. 9 (6), 4283–4293. 10.21037/apm-20-967 33183058

[B103] Xie W.-Q. Xiao W.-F. Tang K. Wu Y.-X. Hu P.-W. Li Y.-S. (2020b). Caloric Restriction: Implications for Sarcopenia and Potential Mechanisms. Aging 12 (23), 24441–24452. 10.18632/aging.103987 33226962 PMC7762489

[B104] Xie W.-Q. He M. Yu D.-J. Wu Y.-X. Wang X.-H. Lv S. (2021). Mouse Models of Sarcopenia: Classification and Evaluation. J. Cachexia Sarcopenia Muscle 12 (3), 538–554. 10.1002/jcsm.12709 33951340 PMC8200444

[B105] Yeung S. S. Y. Reijnierse E. M. Pham V. K. Trappenburg M. C. Lim W. K. Meskers C. G. M. (2019). Sarcopenia and its Association with Falls and Fractures in Older Adults: A Systematic Review and Meta‐analysis. J. Cachexia Sarcopenia Muscle 10 (3), 485–500. 10.1002/jcsm.12411 30993881 PMC6596401

[B106] Yin H. Price F. Rudnicki M. A. (2013). Satellite Cells and the Muscle Stem Cell Niche. Physiol. Rev. 93 (1), 23–67. 10.1152/physrev.00043.2011 23303905 PMC4073943

[B107] Zhang H. Ryu D. Wu Y. Gariani K. Wang X. Luan P. (2016). NAD + Repletion Improves Mitochondrial and Stem Cell Function and Enhances Life Span in Mice. Science 352 (6292), 1436–1443. 10.1126/science.aaf2693 27127236

[B108] Zhou J. Freeman T. A. Ahmad F. Shang X. Mangano E. Gao E. (2013). GSK-3α is a central Regulator of Age-Related Pathologies in Mice. J. Clin. Invest. 123 (4), 1821–1832. 10.1172/jci64398 23549082 PMC3613907

